# Management of Childhood Infections in Poorly Planned Urban Settlements in Kampala and Wakiso Districts of Uganda

**DOI:** 10.4269/ajtmh.20-0115

**Published:** 2020-08-31

**Authors:** Anthony K. Mbonye, Phyllis Awor, Miriam Kayendeke, Kristian S. Hansen, Pascal Magnussen, Sian E. Clarke

**Affiliations:** 1 School of Public Health, College of Health Sciences, Makerere University, Kampala, Uganda;; 2 Infectious Diseases Research Collaboration, Kampala, Uganda;; 3 Department of Public Health, Centre for Health Economics and Policy, University of Copenhagen, Copenhagen, Denmark;; 4 Institute for Immunology and Microbiology, Centre for Medical Parasitology, University of Copenhagen, Copenhagen, Denmark;; 5 Department of Disease Control, London School of Hygiene and Tropical Medicine, London, United Kingdom

## Abstract

The main objective of this study was to assess the management of childhood infections in high-density poorly planned urban areas of Kampala and Wakiso districts in Uganda, to develop a strategy to deliver integrated community case management (iCCM) of childhood illness services. A total of 72 private healthcare facilities were surveyed (36 drug shops, eight pharmacies, 27 private clinics, and one herbal clinic); supplemented by focus group discussions with village health teams (VHTs), drug shops, and private clinic providers. The majority of drug shops (96.4%, 27/28), pharmacies (100%, 8/8), and (68%, private clinics 17/27) were registered; however, supervision was poor. The majority of patients (> 77%) who visited private health facilities were children aged < 5 years. Furthermore, over 80% (29/64) of the children with uncomplicated malaria were reported to have been given artemether–lumefantrine, and 42% with difficulty breathing were given an antibiotic. Although > 72% providers said they referred children with severe illnesses, taking up referral was complicated by poverty, long distances, and the perception that there were inadequate drugs at referral facilities. Less than 38% of all the facilities had malaria treatment guidelines; < 15% had iCCM guidelines; 6% of the drug shops had iCCM guidelines; and < 13% of the facilities had pneumonia and diarrhea treatment guidelines. Village health teams existed in the study areas, although they had little knowledge on causes and prevention of pneumonia. In conclusion, this study found that quality of care was poor and introduction of iCCM delivered through VHTs, drug shops, and private clinics may, with proper training and support, be a feasible intervention to improve care.

## INTRODUCTION

In Uganda, malaria, pneumonia, and diarrhea remain major causes of child morbidity and mortality; yet if diagnosis and treatment are available, most child deaths can be prevented. In 2012, the WHO/UNICEF recommended the implementation of integrated community case management (iCCM) by community health workers (CHWs), to increase access to health care in underserved populations for these three childhood diseases,^1,2^ and most African countries, including Uganda, have begun to scale up this approach.

However, there remain many operational constraints to scaling up this intervention, and there is insufficient evidence at present to recommend a deployment strategy in urbanized settings.^2^ Few studies have been undertaken in urban areas, and the limited evidence available has shown low utilization of CHWs in such settings.^3–6^ Furthermore, the social cohesion required to support CHW volunteers in urban areas is weaker than that in rural communities. Rapidly developing urban settlements also lag behind in terms of public infrastructure; and health systems may have limited capacity to support CHWs. Even in planned urban settlements with a well-established formal and informal private sectors, it has been shown that treatments received in these areas are often inadequate or inappropriate.^3–11^ Training of accredited private sector providers to deliver iCCM remains an alternative approach that has yet to be tested—but has so far been identified as a priority for research.^2–4^

In Uganda, health services in urban and peri-urban areas are provided by licensed drug shops that are regulated by the National Drug Authority (NDA) and licensed to sell only class-C drugs (over-the-counter prescriptions).^7–12^ In addition, there are CHWs who provide primary healthcare services^12–14^ and numerous private clinics that offer curative services and provide referral to higher level health facilities. These private clinics together with drug shops operate under a public–private policy that allows collaboration between the private health facilities and the district health team (DHT).^15^

Thus, studies that generate insights of immediate local relevance on how to improve access to health care in urban areas, inform current debates on the role of the private sector in healthcare provision in Uganda, and help shape models for future implementation are urgently required. Results from such studies would fill an important gap in evidence for policy development, and they would respond to a call by international and national developments for the control of childhood illnesses.^1,2,16,17^

The present study was informed by findings from recent research conducted in registered drug shops in rural Uganda. First, a randomized intervention trial to improve malaria treatment practices in registered drug shops in Mukono district, Uganda,^18–23^ which demonstrated good adherence to treatment guidelines, was conducted. Over 90% of treatment decisions by drug vendors were consistent with malaria rapid diagnostic test (mRDT) results and treatment algorithm, resulting in a substantial improvement in the appropriate targeting of artemisinin combination therapies (ACTs) for malaria from 30% to 70%.^22^ Qualitative evaluation of the intervention suggested a perceived increase in professional status, patient numbers, and profitability, as well as supervisory visits by the Ministry of Health, and reduced fear of closure among drug shop vendors.^21,23^ In addition, it may have been important in promoting adherence to clinical guidelines by vendors. Patients perceived vendors’ skills to be improved, and drug shops reported increases in utilization. A second study examined the feasibility of introducing iCCM to treat malaria, pneumonia, and diarrhea in drug shops in two rural districts of Uganda and documented significant improvement in treatment of children diagnosed with and treated for these diseases.^24^

Based on this evidence, we investigated the feasibility of implementing iCCM in high-density poorly planned urban areas to improve diagnosis and treatment of three major childhood illnesses.^13^ In particular, this study aimed to address the current lack of evidence to inform best practice for delivering health care in urban areas, and to improve access to treatment for the three iCCM target diseases such as malaria, pneumonia, and diarrhea.

The focus on urban areas is because Uganda has experienced a rapid growth in its’ urban population from 18% in 2007 to 23% in 2017; Kampala, the capital city, alone has an annual growth rate of 5.6%.^25^ This rapid urban growth generates major socioeconomic and environmental problems that lower the quality of life of urban dwellers.

Although prior research in Uganda has found that quality of care is often inappropriate in drug outlets in rural areas, it is unknown whether this also applies to private sector drug shops and clinics in urban settings. Thus, our study focused on quality of treatment services, with the aim to develop a strategy to deliver iCCM services for childhood infections in high-density poorly planned urban settings in Kampala and Wakiso districts.

## MATERIALS AND METHODS

### Study setting.

The study was carried out in poorly planned high-density residential areas of Kampala and Wakiso districts in central Uganda. The areas suffer from poorly planned urban expansion around the capital city of Kampala. The majority of the population in Kampala and Wakiso are within a 5-km distance from a treatment facility. A recent survey revealed that the prevalence of malaria among children less than 5 years was below 1% for Kampala and < 5% for Wakiso district.^25^ However, there was a high prevalence of children with diarrhea and pneumonia as shown in a previous study, with a total of 9% of children younger than < 5 years in Uganda showing symptoms of an acute respiratory tract infections 33% had fever, and 20% experienced an episode of diarrhea.^7^

### Study design.

The study had a formative design with the aim to adapt iCCM to poorly planned urban settings. As such, it used the following qualitative data collection methods: 1) semi-structured interviews were carried out within the study area with key informants to characterize and map current sources of treatment available to patients living in a typical high-density poorly planned urban areas in Kampala and Wakiso districts, as well as to collect data on the status of registration, drugs and supplies stocked, availability and knowledge of treatment guidelines, patient volumes, treatment and referral practices, and professional linkages with the health system and other health workers; 2) focus group discussions (FGDs) with a focus on quality of care (availability of staff, level of qualification, availability of drugs and diagnostic equipment, and attitude of staff); and their views on improving quality of care in the private health sector.

Quality of care was defined as availability of childhood services for each level of health facility according to the iCCM guidelines.^13,14^ The study also explored the feasibility of two iCCM delivery strategies—CHWs and private sector providers—in improving access to childcare and whether any of the strategies were acceptable to the local population and national health authorities.

### Sampling and data collection.

A survey to map out sources of treatment available to patients living in the study areas was conducted from October 17, 2016 to November 7, 2016. A snowball sampling approach was used to identify all sources of treatment within each enumeration area. Sampling was continued in adjacent enumeration areas until a minimum of 50 treatment providers had been identified, mapped, and interviewed. It was anticipated that the study area was likely to consist of 2–4 adjacent enumeration areas within a zone of high population density, such as Kinawataka, Kyengera, or Katwe. In total, there were 10 enumeration areas.

Semi-structured interviews with drug shop vendors and private clinics captured data on the treatments usually prescribed for malaria, pneumonia, and diarrhea in children; availability of drugs and diagnostic tests; availability of guidelines and treatment algorithms; providers’ knowledge of treatment guidelines; and professional linkages with the health system and other health workers.

Focus group discussions were undertaken from November 17, 2016 to December 14, 2016 to characterize the context in which the iCCM pilot would be situated; to better define what private facilities were and what they represented for providers, consumers, and other stakeholders within the health system; and perceptions of the quality of health services available in the study area. Focus group discussions also explored the feasibility of a CHW-based model compared with a drug shop model, including potential mechanisms for sustaining CHWs in an urban environment, training and professional development, accreditation, logistics and supply chain management, and reporting systems. A total of 11 FGDs were held: three with members of village health teams (VHTs), two with drug shops, two with private clinics, two with primary care givers, and two with household heads (see Table 1). Participants were selected as follows: there were seven villages in Kyengera (Wakismese, Nkokonjeru A, Nkokonjeru B, Kabojja B, Kyengera Central, Masanda, and Nabazziza) and six villages in Katwe (Bagirinya, Kasule, Lufula, Muwanga, Muwonge, and White Nile). From each village, two drug shop vendors were randomly selected to participate in the FGDs. Two VHTs were purposively selected from each village depending on their previous training in integrated community case management (iCCM). We then relied on VHTs to conveniently select two household heads and primary caregivers of children less than 5 years, respectively, from each of the villages to participate in the FGDs. Focus group discussions were held in an area that ensured privacy. The FGDs were conducted by a graduate social scientist recording the proceedings after obtaining consent.

**Table 1:** Methods and intended outcomes

Method	Research questions	Participants	Data collected	Outcomes synthesized
Semi-structured interviews (72 private healthcare facilities included in survey)	What is type of health care for childhood illnesses in high-density poorly planned urban areas?	36 drug shops, eight pharmacies, 27 private clinics, and one herbal clinic	Status of registration, volume and range of childcare services, availability of drug supplies, and guidelines	Availability and volume of essential health services to manage childhood illnesses were identified
				Factors influencing quality of providing these services in poorly planned urban areas were documented
FGDs (11 FGDs were conducted)	What is the quality of health care for childhood illnesses in high-density poorly planned urban areas?	Three VHTs, two drug shops, two private clinics, two primary caregivers and two household heads	Perceptions on access to care, quality of care and ways of improving care, and feasibility of VHTs and private clinics delivering iCCM	Perceptions of quality of health services and ways of improving quality of care in the study area were assessed
	What is the feasibility of delivering iCCM strategies through CHWs and private sector providers to improve access to childcare?			Ways of improving access to health care in urban areas
	What are the views of key stakeholders in improving quality of care in high-density poorly planned urban areas?			Models for future implementation of iCCM
				How to support CHW volunteers in rural communities?
				Whether private sector providers can deliver iCCM?
Literature review	What is the treatment landscape of childhood illnesses in high-density poorly planned urban areas?	–	Literature on childhood illnesses, existing care and the context of treatment seeking in poorly planned urban areas	The context of health services provision in poorly planned urban areas

DDI = district drug inspector; CHW = community health worker; FDG = focus group discussion; iCCM = integrated community case management; VHTs = village health teams.

### Data management and analyses.

Survey data were captured and archived. Those were then analyzed using STATA version 14.0 (STATA Corporation, College Station, TX). Data from FGDs were initially coded by topics and ideas and, later, grouped into overarching themes using NVivo qualitative data coding software, and results were thematically organized and illustrated using direct quotes.

## RESULTS

### Characteristics of private health facilities.

In total, 72 private healthcare facilities were surveyed. The response rate was 96%, and the reason for nonresponse was refusal of the drug sellers in the health facility, citing lack of authorization from the owner of the facility. The majority of them, 50% (36/72), were drug shops, 37.5% (27/72) were private clinics, and 11.1% (8/72) were pharmacies. One herbal clinic was not registered with any authority. Of those interviewed at health facilities, 27.8% (20/72) owned the facilities (Table 2).

**Table 2:** Characteristics of private health facilities

Characteristic	Drug shops (*N* = 36)	Pharmacies (*N* = 8)	Private clinics (*N* = 27)
Is facility registered	28 (77.8%)	8 (100%)	25 (92.6%)
Body registering facility			
DHT	3 (10.7%)	0	4 (16.0%)
National Drug Authority	24 (85.7%)	7 (87.5%)	4 (16.0%)
Pharmacy council	0	1 (12.5%)	0
Other (UMDPC, AHPC, and UNMC)	1 (3.6%)	0	17 (68.0%)
Years in operation (median)	2	3	5
What is the busiest time at this facility?			
Morning (up to 12 pm)	12 (33.4%)	1 (12.5%)	8 (29.6%)
Afternoon (12–5 pm)	7 (19.4%)	2 (25.0%)	4 (14.8%)
Evening (5–7 pm)	13 (36.1%)	4 (50.0%)	14 (51.9%)
Night (7 pm–12 am)	4 (11.1%)	1 (12.5%)	1 (3.7%)
Open after 7 pm	25 (69.4%)	7 (87.5%)	27 (100%)
Facilities with a patient register	7 (27.8%)	4 (50.0%)	24 (88.9%)
Facilities with stock control register	22 (61.1%)	7 (87.5%)	12 (44.4%)
Level of care of nearest public health facility			
Regional referral hospital	0	0	1 (3.7%)
District hospital	0	0	1 (3.7%)
HCIV	4 (11.1%)	5 (62.5%)	8 (29.6%)
HCIII	17 (47.2%)	0	10 (37.0%)
HCII	15 (41.7%)	3 (37.5%)	7 (26.0%)
Time (minutes) walking to reach the nearest public health facility	22	21	21
Average number of other facilities near this one (10-minute walk)	4	6	8
When (weeks) was the facility last visited by DDI/DHT?*	66	45	58
When (weeks) did you last have professional contact with someone from the nearest health facility?†	40	39	42

DDI = district drug inspector;

DHT = district health team.

* Median 61 weeks.

† Median 40 weeks.

Of the drug shops and pharmacies, > 85.7% were registered with the NDA, whereas 68% (17/27) of the private clinics were registered with the Uganda Medical and Dental Practitioners Council. The facilities had been in operation for an average of 2–5 years. Most of the facilities were busiest in the evenings, and 81.9% were open after 7 pm. The busiest time for sick children (all facilities) was in the morning (31.9%), evening (20.8%), and afternoon (4.2%) (data not shown).

Of the private clinics, 88.9% (24/27) had patient registers, but only 27.8% (7/36) drug shops, and 50% (4/8) of the pharmacies had patient registers (Table 2).

Less than 4% (3/72) of the facilities were near a district or a regional referral hospital (a walking distance of less than 30 minutes), whereas 89.4% (32/36) of the drugs shops and 63% (17/27) private clinics were near health center II or III. Furthermore, it took over a year (66 weeks) for a member of the DHT to visit drug shops, 45 weeks for pharmacies, and 58 weeks for private clinics. Similarly, it took 40 weeks for providers at drug shops to make professional contact with colleagues at the nearest facilities, and it took 39 weeks for pharmacists and 42 weeks for providers at private clinics (Table 2).

### Staff characteristics.

A total of 72 providers were interviewed: 50% at drug shops, 37.5% at private clinics, and 11.1% at pharmacies. The majority of providers were female: 80.6% (29/36) at drug shops, 87.5% (7/8) at pharmacies, and 63% (17/27) at private clinics. Over 75% had tertiary and university education, and this varied across facilities (*P* = 0.006) (Table 3). At drug shops and pharmacies, enrolled nurses were in the majority, with 52.8% (19/36) and 75.0% (6/8), respectively. At private clinics, there were 11.1% (3/27) doctors, 22.2% (6/27) clinical officers, and 40.7% (11/27) enrolled nurses (*P* = 0.004). Providers at drug shops had a longer professional experience, with an average of 8 years compared with 3 years at pharmacies and 7 years at private clinics (*P* = 0.003) (Table 3).

**Table 3:** Staff characteristics at private health facilities

				
	Drug shops (*N* = 36)	Pharmacies (*N* = 8)	Private clinics (*N* = 27)	*P*-value
Gender				
Male	7 (19.4%)	1 (12.5%)	10 (37.0%)	0.006
Female	29 (80.6%)	7 (87.5%)	17 (63.0%)	
Education				
Secondary	2 (5.6%)	0	2 (7.4%)	0.006
Tertiary (certificate/diploma)	27 (75.0%)	8 (100)	22 (81.5%)	
University	1 (2.8%)	0	3 (11.1%)	
Other	6 (16.6%)	0	0	
Level of professional training				
Doctor	0	0	3 (11.1%)	0.004
Clinical officer	0	0	6 (22.2%)	
Enrolled nurse	19 (52.8%)	6 (75.0%)	11 (40.7%)	
Registered nurse	3 (8.3%)	0	0	
Nursing assistant/nursing aide	8 (19.4%)	1 (12.5%)	3 (11.1%)	
Years of professional experience (median years)	8	3	7	0.003

### Availability of drugs and treatment guidelines.

Half of drug shops 50% (18/36), 75% (6/8) of the pharmacies, and 48.2% (13/27) of the private clinics had treatment guidelines or any reference materials. Less than 38% (27/72) of all the private facilities had malaria treatment guidelines, compared with the lowest at private clinics with 7.4% (2/27) and drug shops 15.4% (16/36). Similarly, less than 15% (11/72) of all facilities had integrated management of childhood illness (IMCI) guidelines, and only 5.6% (2/36) of the drug shops had iCCM guidelines. In addition, less than 13% (9/72) of all the facilities had pneumonia and diarrhea treatment guidelines.

Almost all health facilities stocked antimalarial drugs (> 80%). Malaria rapid diagnostic tests were available at 52.8% (19/36) of the drug shops, 50% (4/8) at pharmacies, and 85.2% (23/27) at private clinics. The antibiotics stocked at drug shops ranged from amoxicillin with 58.3% (21/36), any other antibiotic with 52.8% (19/36), oral rehydration salts (ORS) with 83.3% (30/36), and zinc with 80.6% (29/36). At pharmacies and private clinics, these drugs were fully stocked (100%) (Table 4).

**Table 4:** Availability of drugs and treatment guidelines at private health facilities

	Drug shops (*N* = 36)	Pharmacies (*N* = 8)	Private clinics (*N* = 27)
Treatment guidelines/algorithms			
Availability of any treatment guideline or any other reference material	18 (50.0%)	6 (75.0%)	13 (48.2%)
Uganda National Clinical Guidelines	16 (45.4%)	3 (37.5%)	17 (63.0%)
Malaria treatment guidelines	16 (15.4%)	3 (37.5%)	2 (7.4%)
Integrated management of childhood illnesses	0	1 (12.5%)	4 (14.8%)
Integrated community case management	2 (5.6%)	0	0
Malaria treatment charts/algorithms	6 (16.7%)	1 (12.5%)	6 (22.20%)
Pneumonia treatment charts/algorithms	3 (8.3%)	1 (12.5%)	3 (11.1%)
Diarrhea treatment charts/algorithms	4 (11.1%)	1 (12.5%)	3 (11.1%)
Drugs stocked/sold			
Antimalarial drugs	29 (80.6%)	8 (100%)	27 (100%)
Malaria rapid diagnostic tests	19 (52.8%)	4 (50%)	23 (85.2%)
Amoxicillin	21 (58.3%)	8 (100%)	27 (100%)
Any other antibiotic	19 (52.8%)	8 (100%)	27 (100%)
Oral rehydration salts	30 (83.3%)	8 (100%)	27 (100%)
Zinc	29 (80.6%)	8 (100%)	27 (100%)

### Treatment and referral of children.

Many patients (all ages) visited private health facilities a day before the survey, with 83 at drug shops, 143 at pharmacies, and 77 at private clinics. Of these children < 5 years, 75.9% (64/83) were seen at drug shops, 82.5% (118/143) at pharmacies, and 89.6% (69/77) at private clinics. Over 80% (29/64) of the children with uncomplicated malaria (defined as symptomatic malaria with fever and parasitemia but without signs of severe malaria)^13^ were given ACT according to treatment guidelines.^14^ Only 10 children with severe malaria were seen at these facilities, and < 45% were given injectable artesunate as recommended. In addition, 33.3% (12/36) children with cough seen at drug shops were given a cough-suppressant syrup, and those with difficult breathing (41.7%, 15/36) were given an antibiotic. Over 80% of children with diarrhea were given ORS and zinc at all the facilities (Table 5).

**Table 5:** Treatment and referral of children at private health facilities

	Drug shops (*N* = 36)	Pharmacies (*N* = 8)	Private clinics (*N* = 27)
Total number of patients seen a day before the survey	83	143	77
Number of children < 5 years seen a day before the survey	64 (77.1%)	118 (85.2%)	69 (89.6%)
Providers who reported that they give artemisinin-based combination therapy to children with uncomplicated malaria (*N* = 64)	29 (80.6%)	8 (100%)	27 (100%)
Providers who reported that they give injectable artesunate to children with severe malaria (*N* = 44)	14 (38.9%)	3 (37.5%)	27 (100%)
Providers who reported that they give syrup and advice on further management to children with cough (*N* = 44)	21 (58.3%)	6 (75.0%)	17 (63.0%)
Providers who reported that they give amoxicillin to children with breathing difficulties (*N* = 64)	29 (80.6%)	8 (100%)	27 (100%)
Providers who reported that they give oral rehydration salts and zinc to children with diarrhea (*N* = 63)	29 (80.6%)	7 (87.5%)	27 (100%)
Do you ever refer children with severe illnesses to get treatment elsewhere?	26 (72.2%)	8 (100%)	19 (70.4%)
Do you ever send febrile patients elsewhere for laboratory diagnosis?	22 (61.1%)	6 (75.0%)	16 (59.3%)
Where sick children are referred to?			
Private clinic	7 (19.4%)	2 (25.0%)	2 (25.0%)
Health center	9 (25.0%)	2 (25.0%)	6 (75.0%)
Hospital	10 (27.8%)	3 (37.5%)	15 (55.6%)
Do you usually write a referral note when referring children with severe illnesses?	9 (25.0%)	2 (25.0%)	19 (70.4%)
What constraints do you usually encounter in referring patients?			
Patients do not comply	8 (22.2%)	1 (12.5%)	3 (37.5%)
Patients do not have money	16 (44.4%)	3 (37.5%)	19 (70.4%)
Referral facilities are too far	4 (11.1%)	2 (25.0%)	8 (29.6%)
Long walking distances	4 (11.1%)	0	5 (18.5%)
No drugs at referral facilities	4 (11.1%)	0	3 (37.5%)
Referral undermines reputation	0	0	1 (12.5%)
Does the shop/private clinic have disposable syringes and needles?	11 (30.6%)	4 (50.0%)	24 (88.9%)
Does the shop/private clinic have a bin for disposal of sharp objects?	9 (25.0%)	4 (50.0%)	24 (88.9%)

The majority of providers (> 72.0%) said they referred children with severe illness, although > 59% said they referred febrile patients elsewhere for laboratory diagnosis. Sick children were referred to health centers, private clinics, and hospitals. And, 25% (9/36) of providers at drug shops and pharmacies said they usually gave a referral note, and a higher proportion (70.4%, 19/27) at private clinics gave accompanying referral notes. Providers reported that they faced several constraints in referring patients, ranging from patients lacking money, to noncompliance, to referral facilities being far too inadequate in terms of drugs.

### Mechanisms for governance and regulation of VHTs.

The VHTs were based at the village level and were said to be useful to the healthcare system. Although they were widely available in urban areas of Kampala and Wakiso districts, they faced challenges of governance and regulation. Our research found that support supervision, monitoring, or regulation was hardly performed and that dedicated supervision was rare and usually offered when there were other ongoing public health campaigns, as highlighted by in the following texts:

We have supervisors; they are about three. They go to the some VHTs and get reports from them, and then bring those reports here to the health facility on a monthly basis, and from there, they are taken to the district. They used to come in the past, especially when they brought us drugs, but they stopped. (Respondent 6, FGD with VHTs, Kyengera)

### Quality assurance of services.

The VHTs are people with a basic educational background and who usually receive short training courses in some specific areas. Our research found that each village possessed at least five VHT members, each specialized in a public health area in which they were trained. The areas of specialization included ICCM, water and sanitation, and immunization. The distribution of roles seemed to concur with the fact that each of them were attached to a nongovernmental organization/government program that supports them financially.

### Linkage with public health system.

Village health teams are often faced with a limited stock of diagnostics and medicines for malaria, deworming drugs, and oral salts. They make verbal referral to the nearest public health facility. Referral was perceived as subjective and reported to be dependent on caretakers’ perception of the referral site, affordability, and time. On many occasions, caregivers resorted to accessing health care at private health facilities for their proximity, quick attention, and flexibility covering medical bills.

### Financial sustainability.

Our findings indicate that the VHTs offered services on a voluntary basis. This needs to be reviewed because voluntarism was not considered sustainable. Village health team participants called for a review of the program through streamlining activities, providing supplies in a timely fashion, and being paid for the work they do, as highlighted in the following texts:

In order to address the challenges VHTs face, Government should make sure that the drugs are available in plenty. An allowance of 250,000 shillings or 300,000 shillings every month should be paid to VHTs. Government has to be fair because there are some [clients] who come after 12.00 noon when the patients have already gone. (Respondent 4, FDG1 with VHTs, Katwe)

### Perceived quality of care at private health facilities.

Discussions in the community revealed that there was perceived poor quality of care at private clinics. They said that there was, however, good customer care at these facilities. They also stated that they created a good linkage directly with communities and their facilities were open all the time, as discussed in the following quotes:

Providers mind and care a lot about children, they carry the child and examine it, they counsel the parents or caregivers of the children, but the quality of health care given depends on the financial status of the patient. (FGD with VHTs, Kyengera)

Providers are available all the time, they behave well because they are paid well by the private facility owners, and they prioritize patients. (FGD with VHTs, Kyengera)

Drugs at private clinics are very expensive and they lack immunization vaccines like measles. (FGD with VHTs, Kyengera)

We create a good bond with the community. We follow up patients after treatment to see how they are faring and get involved in community education, we close late so that clients get services, we do this working in shifts. (FGD drug shops/clinics, Kyengera)

Providers at private health facilities are always available because they operate a business, they care a lot for their patients…they welcome patients in a good way and when the client has no money, they will refer [them] to a government health facility. However, the drugs at private facilities are expensive. (FGD with VHTs, Kyengera)

### Perceived quality of care at public health facilities.

Overall, the perceived quality of care in public health facilities was rated as poor in all discussions. This was attributed to having too few staff, who were overworked and did not have time to attend to patients. Providers were described as rude and corrupt, and they charged unauthorized fees for prescriptions and laboratory tests. Long distances to public health facilities and frequent drug stock-outs were cited as constraints to using public facilities, as the following accounts highlights:

At Kisenyi HC IV where everyone goes, providers require you to buy a prescription book and there is usually no medicine; health workers want to be bribed in order to attend to your sick child quickly. We have heard several times that Kampala Capital City Authority does not pay health workers on time, so they are disgruntled. (FGD with VHTs, Kisenyi)

The government health facilities here look good but the health workers are so corrupt that you have to pay a bribe ranging from 5,000–10,000 shillings to get quick attention for your child. (FGD with VHTs, Kisenyi)

Clients don’t seek care at Kiruddu hospital because the hospital has only one gate where patients enter and dead bodies [are] taken away. They perceive that this brings the incoming patients bad luck. (FGD with VHTs, Kisenyi)

The equipment to test patients are there but the personnel to operate them are few or even unavailable. Some health workers are not qualified to use this equipment and for a patient to access one, you need to pay a bribe for malaria testing. Health workers send patients to the laboratory for blood and stool tests but there are no personnel to carry out the tests. (FGD, Katwe.)

When we refer patients there, they are afraid of the long queues at Kisenyi HC IV and at Kiruddu hospital, providers have poor attitude due to poor working conditions. There are many clients, but not enough manpower, not enough drugs, and delayed salaries. “I receive medical forms from Kisenyi HC IV indicating that they do not have medicine”, “providers only work in the morning hours and they leave interns who cannot handle serious illnesses” (FGD with VHTs, Kisenyi.)

### Knowledge on causes and prevention of malaria, diarrhea, and pneumonia by VHTs.

Overall, people in the study communities knew what caused malaria and diarrhea and how to treat and prevent them. Malaria was thought to be caused by: not closing windows and doors early; not sleeping under a mosquito net; stomach worms; stagnant water; broken bottles; and bushes, where mosquitos breed; and drinking dirty or unboiled water. Additionally, malaria was thought to be treated and prevented in the following ways: treatment with Coartem; sleeping under bed nets; draining stagnant water; closing doors and windows early; spraying the house; and covering toilets to stop mosquitos from breeding (FGD-Kyengera, FGD-Katwe). The causes of diarrhea were thought to be eating dirty and cold food; not covering food and drinks well; drinking unboiled water and releasing full sewage into water sources; not washing hands after visiting the toilet; dirty utensils; house flies; measles; and drinking unboiled water. While the following practices were thought to prevent diarrhea: giving children boiled drinking water; stopping children from playing in dirty trenches sensitizing the community to maintain environmental hygiene; sensitize food vendors to maintain personal and hand hygiene as they serve food to customers; trim children’s finger nails; educating the community on covering food; cleaning feeding bottles for children; constructing a dish stand; deworming children; and taking ORS and zinc tablets (FGD-Kyengera, FGD-Katwe). Overall, there were misconceptions on causes of pneumonia in these two communities, with possible implications for treatment seeking. Pneumonia was thought to be caused by pollution from cars and cigarettes; children who share food from the same plate; people spitting on the ground then dust falls on sweets and children sharing them; cold weather and wind; flu; too much dust; inadequate immunization; air contamination; covering children with wet clothes; prolonged cough and fever. Pneumonia was thought to be prevented by covering children during cold weather, a balanced diet, immediate treatment of children with cough with amoxicillin, immunizing children with diphtheria pertussis tetanus, boiling drinking water, sensitizing parents about keeping children warm, distributing health education charts on prevention of pneumonia (FGD-Kyengera, FGD-Katwe).

### Work of VHTs in poorly planned urban settings.

In the two study areas, VHTs existed. It was reported that most of them had received training from Kampala Capital City Authority, and they were the link to the community, as mentioned in the following quote:

They encourage parents to take sick children for treatment to public health facilities, they sensitize the community about health issues like immunization, they do home visits, check on sick children and encourage them to go for treatment, distribute medicine and sensitize community about hygiene and treatment seeking. (FGD-Katwe)

### Challenges faced by VHTs.

It was reported that VHTs did not have items to use such as gloves, gumboots, uniforms, and medicines. In addition, they needed refresher courses on management of childhood illness and nutrition, and it was agreed that VHTs needed facilitation to enable them do their voluntary work. However, it was noted that VHTs did not receive regular supervision, except when there was an ongoing government health campaign. Finally, it was recommended that VHTs be paid an allowance ranging from 5,000–10,000 shillings weekly since voluntary work was not deemed to be sustainable (FGD-Katwe).

### Feasibility of introducing iCCM in poorly planned urban settings.

Integrated community case management was considered acceptable and recognized as an important intervention to improve child welfare in the study communities. It was, thus, recommended that government needed to collaborate with private health facilities to treat diseases by organizing training workshops for providers at drug shops and private clinics. It was further recommended that government should provide malaria and HIV test kits to private health facilities. Private providers remarked that they wanted government to recognize them, offer training, and have frequent interactions in the form of supervision, as the following responses highlight:

`We need to be recognized by government, we pay a lot of taxes yet they want us to provide services without support. (FGD-Kyengera)

Private health facilities are neglected, instead government extorts a lot of money in the form of taxes, license and sub county fees. (FGD-Katwe)

Government should provide ICCM medicines on time and in enough quantities, the community needs to know the amount of ICCM drugs delivered, their expiry dates and fixed prices at which the drugs will be sold. (FGD-Kyengera)

It was recommended that the government should introduce iCCM in the community by training private providers and VHTs. It was also recommended that the government should provide the ICCM package at no cost to private providers, so that they can subsidize the service to the community.

### Staff professional development.

Few providers at drug shops and pharmacies (< 39%) had heard of IMCI, compared with 55.6% (15/27) at private clinics. Similarly, less than 38% of providers at drug shops and pharmacies belonged to a professional organization compared with 59.3% (16/27) at private clinics. Almost half of the providers had not received any form of training in the year preceding the survey. Furthermore, few providers at drug shops and private clinics had received training on how to recognize signs of severe illnesses in children, signs of severe malaria and pneumonia, how to use mRDTS, and how to manage children following the iCCM guidelines (Table 6).

**Table 6:** Staff professional development at private health facilities

	Drug shops (*N* = 36)	Pharmacies (*N* = 8)	Private clinics (*N* = 27)
Have you ever heard of IMCI?	14 (38.9%)	3 (37.5%)	15 (55.6%)
Are you a member of a professional organization?	8 (22.2%)	3 (37.5%)	16 (59.3%)
In the last 1 year, have you received any training?	16 (44.4%)	6 (75.0%)	13 (48.2%)
Training received on childhood illnesses			
Signs of severe illness	8 (22.2%)	5 (62.5%)	8 (29.6%)
Signs of severe malaria	8 (22.2%)	5 (62.5%)	5 (18.5%)
Signs of pneumonia	3 (8.3%)	2 (25.0%)	3 (11.1%)
Malaria rapid diagnostic tests	8 (22.2%)	3 (37.5%)	3 (11.1%)
IMCI/integrated community case management	5 (13.9%)	1 (12.5%)	1 (3.7%)
Do you have a mobile phone?	27 (75.0%)	7 (87.5%)	24 (88.9%)
Do you have a smartphone?	19 (52.8%)	6 (75.0%)	16 (59.3%)
Do you have access to a computer?	4 (11.1%)	4 (50.0%)	13 (48.2%)
Do you have access to Internet?	17 (47.2%)	6 (75.0%)	17 (63.0%)

IMCI = integrated management of childhood illnesses.

More than 75% of the providers at all facilities had mobile phones, although 52.8% (19/36) at drug shops, 75.0% (6/8) at pharmacies, and 59.3% (16/27) at private clinics had smartphones. A high proportion of providers at pharmacies (50%, 4/8) and private clinics (48.2%, 13/27) had access to computers, compared with 11.1% (4/36) at drug shops. Similarly, over 63% (17/27) of providers at pharmacies and private clinics (47.2%, 17/36) had access to the internet compared with drug shops (Table 6).

## DISCUSSION

This study was conceived to address a major gap in service provision in Uganda’s unplanned urban settlements. The rapid population growth rate in these settlements increases the urgency on generation of consistent evidence regarding effective approaches to deliver health care.^26^ Thus the study aimed to investigate and compare the feasibility of two alternative community-based mechanisms to deliver iCCM services: 1) CHWs and 2) private retail outlets. To achieve this, the study assessed the availability and quality of services for childhood illnesses such as malaria, pneumonia, and diarrhea.

Results show that most of the children with uncomplicated malaria were given artemether–lumefantrine, whereas fewer with difficulty breathing were given an antibiotic. It is noted that referral uptake was complicated by poverty, long distances, and the perception that there were inadequate availability of quality of drugs at referral facilities. In addition, few facilities had malaria treatment guidelines, iCCM guidelines, and pneumonia and diarrhea treatment guidelines.

Overall, the perceived quality of care was poor in relation to the management of childhood diseases, especially pneumonia, and this was attributed to lack of iCCM in the community, few treatment guidelines, and few providers trained in IMCI and iCCM. At drug shops, a large proportion of children with cough were given a cough-suppressant syrup, rather than an antibiotic as the iCCM guideline recommends. Poor quality of care was also reported in public health facilities. The findings of poor quality of health care in the study areas of Kampala and Wakiso districts concur with previous findings that showed inadequate sanitation and water supply in slum areas of Bwaise (Kampala district) predisposed people to diarrhea and other infectious diseases.^27^

The present results show that poorly planned settlements in Kampala and Wakiso districts have numerous private facilities, and the majority are registered with national authorities. In addition, they have CHWs commonly known as VHTs who are involved in several health promotion activities. Results further show that private facilities attend to a large number of patients, and the majority are children. These results show that there was potential to improve treatment of childhood illnesses in this setting because iCCM was acceptable. In addition, VHTs, drug shops, and private clinics recommended that government introduces iCCM through training, supervision, and providing an iCCM package at a subsidized price. There was also opportunity to deliver web-based interventions because the majority of facilities had access to computers and the Internet.

The present results compare favorably with previous findings where urban private facilities were more likely to be registered and have more trained providers with tertiary education.^20–24^ The high proportion of private clinics that had patient registers was a good indicator and a potential to improve quality of care in these facilities. This is also an implication of adherence to the guideline that requires private facilities to keep patient records.

The study notes a high proportion of drug shops and private clinics with mRDTs; this is because of the current malaria control policy, which allows use of mRDTs at a community level, and there was a high level of community awareness of diagnosis of malaria before treatment. This may explain why a high proportion of children with uncomplicated malaria were given artemether–lumefantrine (Coartem).

It has been shown that overcrowding in poor settlements (slums) contributes to a high prevalence of tuberculosis and food insecurity and that the failure of many national governments to identify slums as distinct from other urban areas prevents the effective implementation of health improvement interventions in slums. Only by making this change and recognizing that slums are a modern and growing phenomenon with a set of health challenges and directing research and resources to address these can be real progress to be made.^28^

A study in India comparing height for age among children showed that the welfare of slum children was significantly worse compared with their rural counterparts. The stunting of children was attributed to slum conditions, such as poor nutrition, repeated infections, and overcrowding.^29^ In addition, a study in Jordan revealed that slum residents suffered from many challenges such as severe poverty; unemployment; illiteracy; low education; gender discrimination; insufficient and poor diet; social and official exclusion; unhealthy environment; lack of water supply, electricity, and basic sanitation facilities; high prevalence of diseases; and insufficient and inappropriate health services.^30^

These present results confirm our hypothesis that poorly planned poor urban settlements in Kampala and Wakiso districts are at disadvantaged when it comes to the provision of health care to children. Uganda is currently experiencing a high rate of urbanization, and populations living in poorly planned, overcrowded areas are likely to be vulnerable to diseases. Because children are a vulnerable group, prioritizing interventions targeting them will help Uganda achieve universal access to health care and reduce the overall burden of disease.

It has been previously shown in the study area that iCCM was perceived to facilitate timely access to treatment and improved child health in peri-urban settings. In addition, caregivers valued VHTs’ free, proximal services, caring attitudes, perceived treatment quality, perceived competency and protocol use, and follow-up and referral services^31^ a justification to scale up iCCM to these areas.

Previous studies in Uganda have shown that training and supervising drug shops improve malaria diagnosis and treatment.^24^ It has also been shown in Uganda that iCCM introduced in the private sector led to increasing community awareness. The intervention improved prompt care seeking within 24 hours of onset of symptoms, included a high adherence to mRDT results, and appropriate treatment of mRDT-positive children with ACTs was high, as well as diagnosis and treatment of diarrhea and pneumonia.^11^ This provides further justification to scale up iCCM in poorly planned settlements in Uganda.

The study also aimed at exploring mechanisms for governance, quality assurance of services, regulation, linkage with public health system, and financial sustainability. Our findings show that because the community was aware, it was the government’s responsibility to introduce iCCM; the present results need to be discussed with the district and national policy-makers to identify modalities of introducing iCCM in slum areas of Kampala and Wakiso districts. Scaling up iCCM in these poorly planned urban areas would be documented, and lessons were used to scale it up to similar settings across the country. It has been shown that over > 50% of the providers owned smartphones and had access to a computers and Internet, thus providing opportunity to deliver web-based iCCM and other professional updates.

The implications of the present results are that iCCM should be introduced at a community level by training VHT members. As they have little knowledge on the causes of diseases and how they can be prevented, the iCCM curriculum would have to be modified to allow for a longer duration of training and follow-up. Although drug shop providers had adequate knowledge on childhood diseases and they said they refer most sick patients for better management, introducing iCCM to drug shops will improve diagnosis, management, and referral of child infections.

The next steps are to design a new intervention strategy to improve access to treatment for children living in rapidly expanding peri-urban areas in Uganda, which will be tested preferably by a cluster-randomized study to assess the cost-effectiveness of iCCM delivery in poorly planned urban areas. The design of the study will be finalized in consultation with national and local public health staff, private providers, and community representatives.

While interpreting our results, we are aware that the study was qualitative in nature and that we were unable to assess the prevalence of childhood diseases and their determinants. We also did not assess the perceptions of quality of care from the care seekers, thus limiting our findings to perceptions of only providers and community and opinion leaders. Nonetheless, some of the opinion and community leaders could have been care seekers themselves. Other studies to assess these aspects, the local epidemiology, and health seeking patterns in poorly planned urban settings are needed to help design future health interventions.

We also aimed to interview drug shops and representatives of the local community to inform the future pricing of iCCM treatment fees at registered drug shops, but this was not explored. We would have known whether the price reflects iCCM as a package of services—charged at one overall fixed price for the whole package, irrespective of treatments received—or if diagnostic services and drugs should be priced individually such that total expenditure to customers will depend on actual tests performed and drugs offered. We recommend these issues to be explored in future studies.

In conclusion, we have shown that there was perceived poor quality of care given to treat childhood illnesses in private health facilities in poorly planned areas of Kampala and Wakiso districts. The study also identified numerous providers ranging from VHTs, drug shops, private clinics, and public health facilities. Furthermore, several health system constraints were identified that limit effective treatment and referral of patients. Finally, the study revealed that introducing iCCM was acceptable and feasible through training and facilitating VHTs, drug shops, and private clinics.

## WHAT THE STUDY ADDS TO THE LITERATURE

**Table:** 

What is already known about this subject?
Delivery and quality of childcare services in rural and planned urban areas in Uganda and elsewhere are well described. However, studies focusing on poorly planned urban areas are scant.
What does this study add?
Data presented in this study show that there was perceived poor quality of care for childhood illnesses in poorly planned urban settings. Introducing iCCM was acceptable and feasible through training and facilitating VHTs, drug shops, and private clinics linked to the public health system with government leadership.
How might this impact on policy and practice?
Scaling up this intervention will contribute to reducing morbidity and mortality among children in poorly planned urban settings in Uganda and contribute to the aspiration of achieving universal health access.
